# An exploration of the correlations between seven psychiatric disorders and the risks of breast cancer, breast benign tumors and breast inflammatory diseases: Mendelian randomization analyses

**DOI:** 10.3389/fpsyt.2023.1179562

**Published:** 2023-06-28

**Authors:** Fei Ren, Qingyao Shang, Shuangtao Zhao, Chenxuan Yang, Kexin Feng, Jiaxiang Liu, Xiyu Kang, Ruixuan Zhang, Xiang Wang, Xin Wang

**Affiliations:** ^1^Department of Breast Surgical Oncology, National Cancer Center/National Clinical Research Center for Cancer/Cancer Hospital, Chinese Academy of Medical Sciences and Peking Union Medical College, Beijing, China; ^2^Department of Thoracic Surgery, Beijing Tuberculosis and Thoracic Tumor Research Institute/Beijing Chest Hospital, Capital Medical University, Beijing, China; ^3^Peking Union Medical College, Beijing, China

**Keywords:** mendelian randomization, psychiatric disorders, breast cancer, breast benign tumors, breast inflammatory diseases

## Abstract

**Background:**

Previous observational studies have showed that certain psychiatric disorders may be linked to breast cancer risk, there is, however, little understanding of relationships between mental disorders and a variety of breast diseases. This study aims to investigate if mental disorders influence the risks of overall breast cancer, the two subtypes of breast cancer (ER+ and ER-), breast benign tumors and breast inflammatory diseases.

**Methods:**

During our research, genome-wide association study (GWAS) data for seven psychiatric disorders (schizophrenia, major depressive disorder, bipolar disorder, post-traumatic stress disorder, panic disorder, obsessive-compulsive disorder and anorexia nervosa) from the Psychiatric Genomics Consortium (PGC) and the UK Biobank were selected, and single-nucleotide polymorphisms (SNPs) significantly linked to these mental disorders were identified as instrumental variables. GWAS data for breast diseases came from the Breast Cancer Association Consortium (BCAC) as well as the FinnGen consortium. We performed two-sample Mendelian randomization (MR) analyses and multivariable MR analyses to assess these SNPs’ effects on various breast diseases. Both heterogeneity and pleiotropy were evaluated by sensitivity analyses.

**Results:**

When the GWAS data of psychiatric disorders were derived from the PGC, our research found that schizophrenia significantly increased the risks of overall breast cancer (two-sample MR: OR 1.05, 95%CI [1.03-1.07], *p* = 3.84 × 10^−6^; multivariable MR: OR 1.06, 95%CI [1.04-1.09], *p* = 2.34 × 10^−6^), ER+ (OR 1.05, 95%CI [1.02-1.07], *p* = 5.94 × 10^−5^) and ER- (two-sample MR: OR 1.04, 95%CI [1.01-1.07], *p* = 0.006; multivariable MR: OR 1.06, 95%CI [1.02-1.10], *p* = 0.001) breast cancer. Nevertheless, major depressive disorder only showed significant positive association with overall breast cancer (OR 1.12, 95%CI [1.04-1.20], *p* = 0.003) according to the two-sample MR analysis, but not in the multivariable MR analysis. In regards to the remainder of the mental illnesses and breast diseases, there were no significant correlations. While as for the data from the UK Biobank, schizophrenia did not significantly increase the risk of breast cancer.

**Conclusions:**

The correlation between schizophrenia and breast cancer found in this study may be false positive results caused by underlying horizontal pleiotropy, rather than a true cause-and-effect relationship. More prospective studies are still needed to be carried out to determine the definitive links between mental illnesses and breast diseases.

## 1. Introduction

Breast illnesses commonly consist of malignant tumors, benign tumors, inflammatory ailments and so on. Among all breast malignancies, breast cancer has the greatest impact on human beings, the incidence of which has been the highest amongst lady cancers, and it is additionally the leading cause of death among women with cancers (1, 2). Breast benign tumors and inflammatory ailments also affect women’s fitness and qualities of lives to various degrees.

Among the potential elements that may additionally influence risks of breast diseases, mental factors are receiving growing attention (3). Previous researches have linked some mental ailments to an multiplied hazard of breast cancer (4, 5). Several observational studies confirmed that major depressive disorder would possibly increase the incidence of breast cancer (6–8). A systematic overview of thirteen researches indicated that sufferers with schizophrenia had an elevated threat of breast cancer (9), with standardized incidence rates (SIR) of 1.11 (1.00–1.22),1.20 (1.05–1.38)and 1.15 (0.98–1.34) in the three largest studies (10–12). To date, however, there were too few sorts of psychiatric disorders covered in preceding studies, and the etiological effects of mental illnesses on breast cancer still lack comprehensive and systematic researches. At the identical time, it has now not been decided whether or not mental sicknesses are associated with the hazards of breast benign ailments or inflammatory diseases. Therefore, greater well-designed researches are expected to elucidate whether there are the cause-and-effect relationships between various psychiatric problems and breast diseases.

Mendelian randomization (MR) is a promising causal reasoning device developed in current years (13, 14), which makes use of genetic variations intently associated with exposures as instrumental variables to discover causal relationships between exposures and outcomes (14–16). The MR analysis is counted on natural random classifications of genetic variations (17, 18). Mendelian inheritance holds that each parent randomly imparts one allele for each gene to its offspring (19). The MR analysis can overcome the risk of bias triggered by using unidentified confounding factors, reverse causality, and dimension errors (4, 20). Therefore, The MR may serve as an alternative to randomized controlled trials (21, 22). In order to perform a MR analysis, it is necessary to satisfy three hypotheses: (1) variations in genetics must be strongly correlated with the exposure; (2) genetic variations must not be affected by any other possible confounding variables whatsoever; (3) genetic instruments only impact the outcome through the exposure (19, 23, 24). Of these, the second and third hypotheses, collectively referred to as the independence of horizontal pleiotropy, can be tested statistically (25).

Two-sample MR and multivariable MR analyses were conducted in this study using genome-wide association study (GWAS) statistics to investigate causal relationships between seven psychiatric disorders and the risks of breast cancer, breast benign tumors, and breast inflammatory diseases. This work can be used to provide insights to further screening or prevention of breast diseases in clinical settings.

## 2. Methods and materials

### 2.1. GWAS data on psychiatric disorders

A total of seven psychiatric disorders were considered as exposures in this study, including schizophrenia, major depressive disorder, bipolar disorder, post-traumatic stress disorder, panic disorder, obsessive–compulsive disorder and anorexia nervosa. We searched the Psychiatric Genomics Consortium (PGC) in https://www.med.unc.edu/pgc/, in order to identify genetic variations connected with these psychiatric disorders among the population of European descent. The number of single nucleotide polymorphisms (SNPs) differed significantly across the GWAS data for each psychiatric disorder, consequently, to make the analyses as accurate as possible, we extracted effect sizes of genetic variations conforming to the genome-wide significance levels (*p* < 5 × 10^−8^) and not falling into linkage disequilibrium (LD *r*^2^ < 0.1, kb = 10,000) when exploring schizophrenia, major depressive disorder and bipolar disorder. Nevertheless, the conditions for selecting instrumental variables were set to *p* < 5 × 10^−6^ and LD *r*^2^ < 0.001 as for the remaining four mental disorders. Several previous analyses also adopted the settings described above (26, 27). Furthermore, to avert biases caused by weak instrumental variables, we determined the variance in phenotype explained by each instrument with R^2^: R^2^ = [2 × EAF × (1-EAF) ×(β)^2^]/[(2 × EAF × (1-EAF) × (β)^2^)+(2 × EAF × (1-EAF) × N ×se (β)^2^)], where EAF was the effector allele frequency, β was the effector size, N was the sample size and se (β) was the standard error of the genetic effect. And then we calculated the F statistic: F = [R^2^ × (N-k-1)]/[(1-R^2^) × k], assessing the strength of the statistics (24, 28, 29), in which k was the number of instrumental variables (16). SNPs with *F* < 10 that were considered weak instrumental variables had been discarded (18). Detailed information regarding the genetic tools selected for each of the seven mental disorders was provided in Supplementary Table S1.

### 2.2. GWAS data on breast diseases

Data on overall breast cancer and two kinds of molecular subtypes (ER+ and ER-) were obtained from the Breast Cancer Association Consortium (BCAC), which were based on GWAS studies of 228,951 women of European descent (122,977 women with breast cancer [69,501 ER+ cases, 21,468 ER-cases] and 105,974 controls) (30). Additionally, the FinnGen consortium supplied GWAS information on breast benign tumors (2079 patients and 101,074 controls) and breast inflammatory diseases (757 patients and 115,030 controls). Data above can be publicly accessible on MRC Integrative Epidemiology Unit.^1^ Detailed information on GWAS data for breast diseases was presented in Supplementary Table S2.

### 2.3. Statistical analyses

The SNPs of mental disorders were extracted from outcome data, excluding those that were strongly associated. Further, we aligned the alleles of SNPs from exposures and outcomes, and then discarded SNPs with incompatible alleles or palindromic SNPs with intermediate allelic frequencies. We also excluded SNPs associated with confounding factors of the exposures and outcomes. Finally, exposures with more than three SNPs were retained for MR analyses. The two-sample MR analyses mainly used inverse variance weighted(IVW), Weighted median and MR Egger to examine the causal relationships between each mental disease and risks of the overall breast cancer, the two subtypes (ER+ and ER−), breast benign tumors and inflammatory diseases. In order to make the conclusions more reliable, we used two methods of multiple tests which called the Bonferroni method and the false discovery rate (FDR) method. The Bonferroni method was more stringent than the FDR method. In the Bonferroni method, we defined that *p* < 0.05/n (n was the number of exposures) was statistically significant, *p* > 0.05 meaned no statistical significances, and 0.05/*n* < *p* < 0.05 represented suggestive statistical significances. While for the FDR method, we obtained adjusted *p* values corrected by the multiple corrections, and *p* < 0.05 was statistically significant.

Heterogeneity tests were carried out using Cochran’s Q statistic, which pointed out that the extent to individual effect size differences among selected genetic variants was due to actual differences among SNPs rather than sampling errors. *p* < 0.05 was recognized as existing heterogeneity (31, 32). When the intercept value deviated from zero, the MR-Egger regression method was applied to identify possible horizontal pleiotropy (33). In addition, we focused on identifying genetic variations in outliers by using MR pleiotropic residuals and outlier tests (MR-PRESSO), and reevaluated the effect estimates once outliers had been removed (34). Finally, leave-one-out (LOO) analyses were performed to determine the robustness of MR estimates as well as whether any associations were driven by particular SNPs.

According to previous researches, MR analyses may result in false positives if genetic correlations occur among exposures (35, 36). Consequently, two methods, multivariable IVW and multivariable MR-Egger, were employed to carry out multivariable MR analyses on major depressive disorder and schizophrenia. Furthermore, we performed linkage disequilibrium score regression (LDSC) to consider the potential genetic correlations between mental disorders and breast cancer. *p* < 0.05 indicated statistical significances. In order to account for confounding effects of sex differences, we also used GWAS data of prostate cancer to evaluate the genetic associations between psychiatric disorders and prostate cancer. Finally, to verify our results, we re-performed MR analyses using GWAS data for seven psychiatric disorders from the UK biobank,^2^ the links of which were indicated in Supplementary Table S3. All analyses were conducted with the statistical software R version 4.2.3 using the “TwoSampleMR,” “data.table,” “ieugwasr,”” plyr” and “MendelianRandomization,” “dplyr,” “gwasvcf,” “VariantAnnotation,” “S4Vectors,” “rio,” “MRlap” packages.

## 3. Results

### 3.1. Relationships between mental disorders and overall breast cancer risk

**Figure 1:**
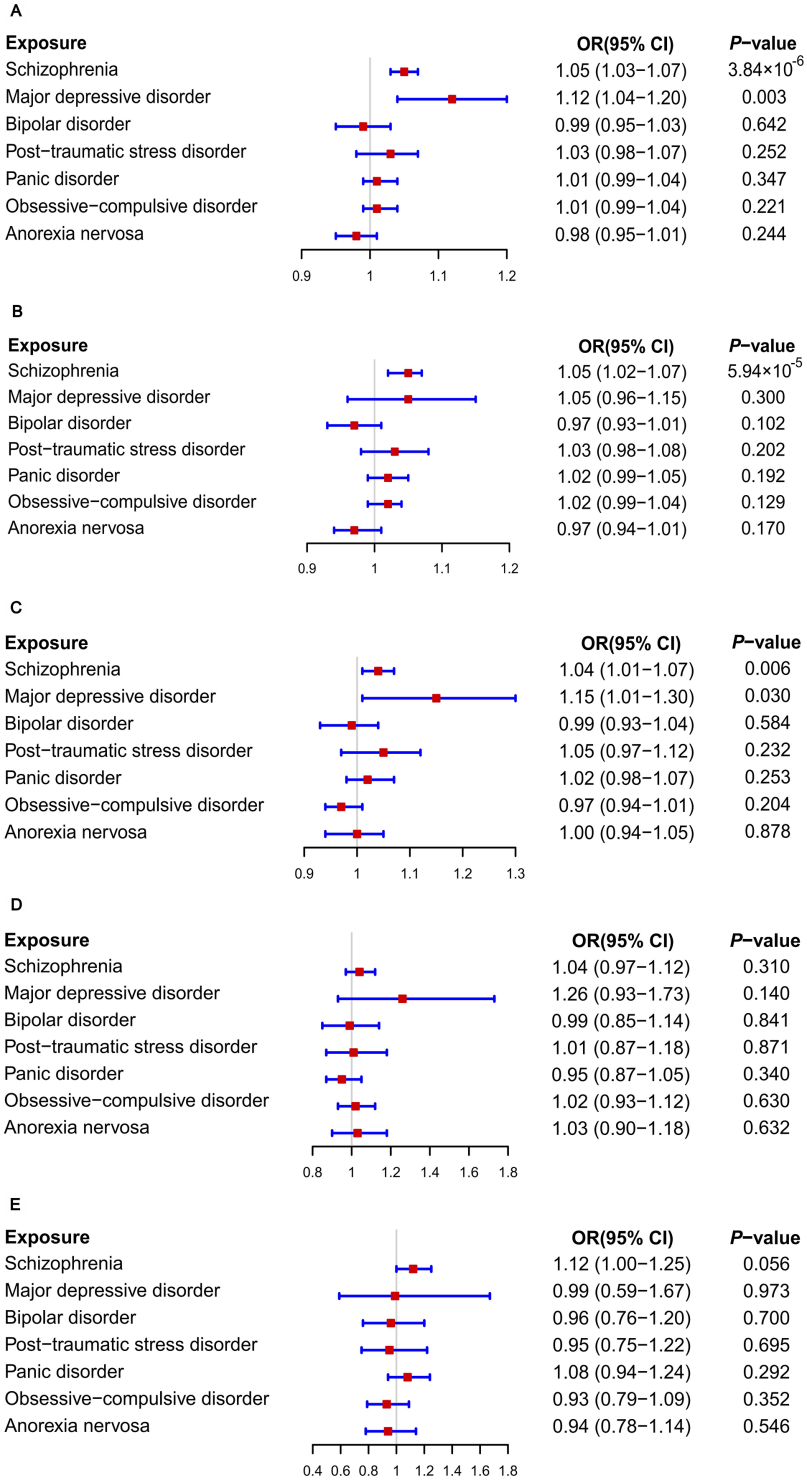
Causal associations between seven psychiatric disorders and the risks of breast diseases: Results of MR analyses using the IVW method. **(A)** Overall breast cancer; **(B)** ER+ breast cancer; **(C)** ER− breast cancer; **(D)** Breast benign tumors; **(E)** Breast inflammatory diseases.

**Figure 2:**
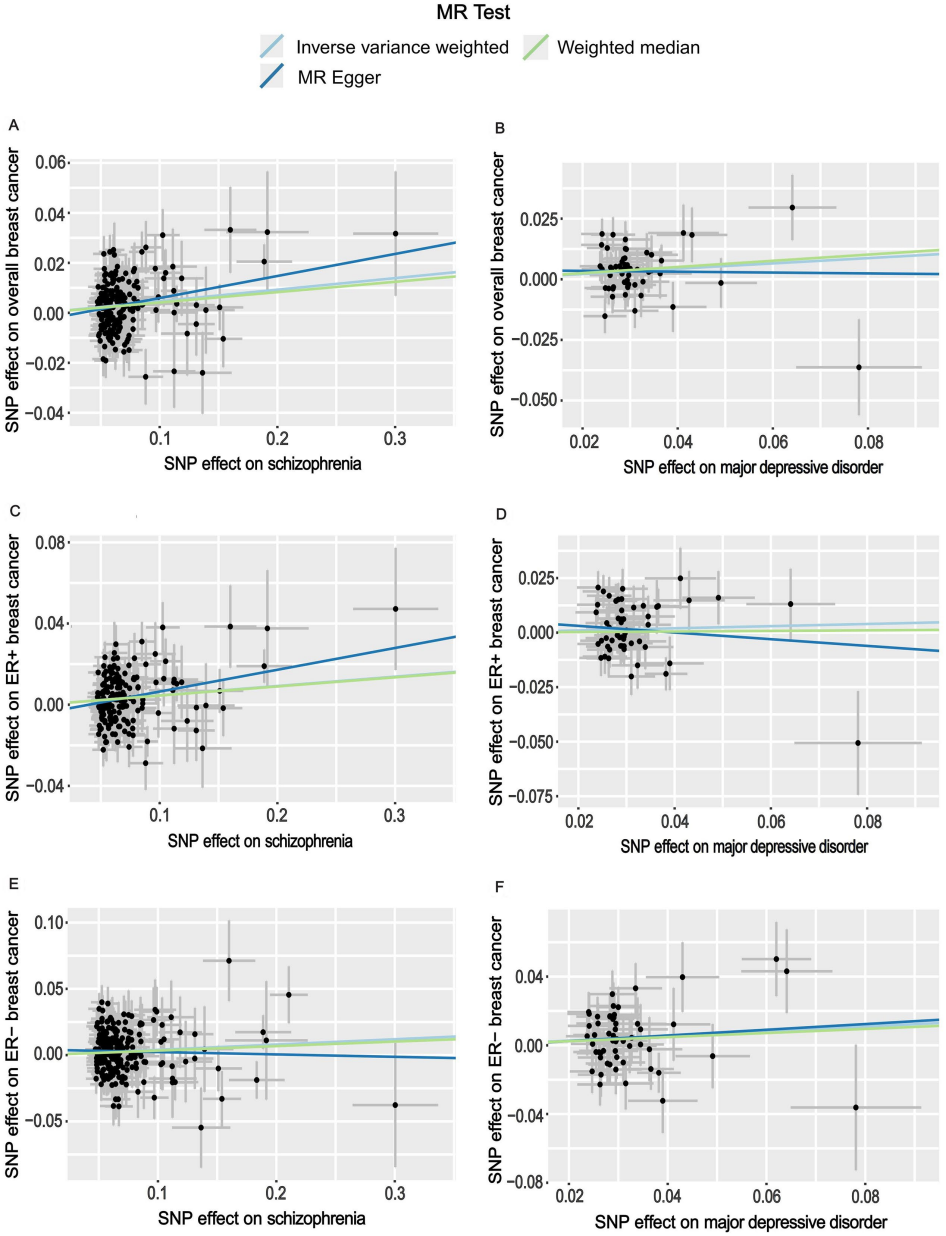
Scatter plots for the effects of schizophrenia and major depressive disorder on the risk of breast cancer. **(A)** Causal relationship between schizophrenia and overall breast cancer; **(B)** Causal relationship between major depressive disorder and overall breast cancer; **(C)** Causal relationship between schizophrenia and ER+ breast cancer; **(D)** Causal relationship between major depressive disorder and ER+ breast cancer; **(E)** Causal relationship between schizophrenia and ER− breast cancer; **(F)** Causal relationship between major depressive disorder and ER− breast cancer.

**Figure 3:**
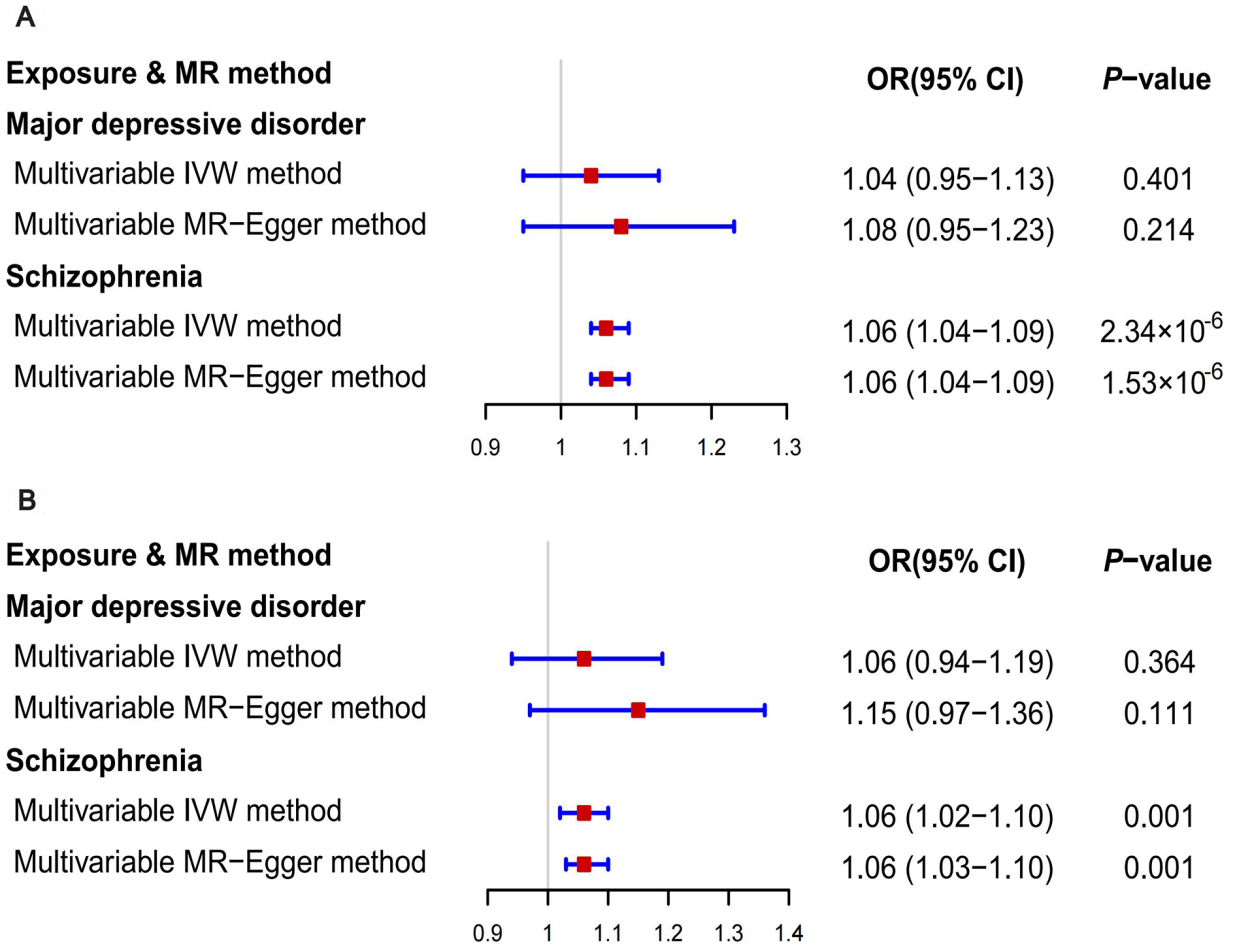
The results of multivariable MR analyses. **(A)** The outcome was overall breast cancer; **(B)** The outcome was ER- breast cancer.

When GWAS data of exposures were derived from the PGC, the two-sample MR analyses using IVW method revealed a significant increase in overall breast cancer risk for individuals with major depressive disorder (OR 1.12, 95%CI [1.04–1.20], *p* = 0.003) and schizophrenia (OR 1.05, 95%CI [1.03–1.07], *p* = 3.84 × 10^−6^) (Figure 1). The results of the two multiple correction methods were consistent. Table 1 showed adjusted *p* values after the multiple corrections using the FDR method. Scatter plots depicted causal estimates derived from each instrumental variable (Figure 2). The estimated correlation values obtained by MR-Egger and Weighted median methods were generally consistent with those calculated through IVW (Table 2). For schizophrenia, there was significant heterogeneity among instrumental variables (*P*_heterogeneity_ < 0.05), but not for major depressive disorder (Table 2). Nevertheless, no clear evidences of horizontal pleiotropy were observed (*P*_pleiotropy_ > 0.05). Based on the leave-one-out analyses, none of the instrumental variables significantly altered the extent of causation between the two psychiatric disorders and overall breast cancer risk (Supplementary Figures S1, S4). On the contrary, bipolar disorder, post-traumatic stress disorder, panic disorder, obsessive–compulsive disorder, and anorexia nervosa had no statistically significant impacts on the risk of overall breast cancer (Figure 1 and Table 2). However, according to the multivariable MR analyses of major depressive disorder and schizophrenia (Figure 3), no significant correlation between major depressive disorder and overall breast cancer risk was found using regardless of multivariable IVW or multivariable MR-egger, while schizophrenia significantly increased the risk (IVW: OR 1.06, 95%CI [1.04–1.09], *p* = 2.34 × 10^−6^; MR-Egger: OR 1.06, 95%CI [1.04–1.09], *p* = 1.53 × 10^−6^). The results of the LDSC regression analyses between mental disorders and overall breast cancer were presented in Supplementary Table S4. Whereas, when using the GWAS data for mental disorders derived from the UK biobank, we found that schizophrenia did not significantly increase the overall breast cancer risk either using the IVW method or the MR Egger method (Table 3).

**Table 1:** The adjusted *p* values after the multiple corrections using the FDR method.

	Overall breast cancer	ER+ breast cancer	ER- breast cancer
Schizophrenia	2.69 × 10^−5^	4.16 × 10^−4^	0.042
Major depressive disorder	0.011	0.300	0.105
Bipolar disorder	0.642	0.236	0.681
Post-traumatic stress disorder	0.353	0.236	0.354
Panic disorder	0.405	0.236	0.354
Obsessive–compulsive disorder	0.353	0.236	0.354
Anorexia nervosa	0.353	0.236	0.878

The adjusted *p* values were obtained based on the *p* values from IVW method.

**Table 2:** The MR analyses of psychiatric disorders and overall breast cancer risk from MR Egger and Weighted median methods.

Psychiatric disorders	Used SNPs	MR Egger		Weighted median		*P*_heterogeneity_	*P*_pleiotropy_
		OR(95% CI)	*p*-value	OR(95% CI)	*p*-value		
Schizophrenia	189	1.09 (1.02–1.17)	1.34 × 10^−2^	1.04 (1.02–1.07)	1.91 × 10^−3^	4.97 × 10^−7^	0.217
Major depressive disorder	55	0.98 (0.66–1.47)	0.933	1.14 (1.04–1.24)	0.006	0.102	0.534
Bipolar disorder	51	0.82 (0.64–1.06)	0.137	1.00 (0.96–1.05)	0.983	7.04 × 10^−7^	0.153
Post-traumatic stress disorder	17	1.03 (0.93–1.13)	0.571	1.04 (0.98–1.10)	0.231	0.461	0.932
Panic disorder	11	1.00 (0.94–1.06)	0.973	1.02 (0.99–1.06)	0.253	0.206	0.584
Obsessive–compulsive disorder	11	1.00 (0.93–1.08)	0.985	1.01 (0.98–1.04)	0.416	0.285	0.680
Anorexia nervosa	10	0.99 (0.91–1.08)	0.782	0.98 (0.94–1.02)	0.382	0.384	0.883

**Table 3:** The *P* values for MR analyses of psychiatric disorders and overall breast cancer risk (GWAS data for exposures were derived from the UK Biobank).

Psychiatric disorders	*p*-value from IVW	*p*-value from MR Egger	Adjusted *p-*value
Schizophrenia	0.684	0.641	0.688
Major depressive disorder	0.007	0.360	0.049
Bipolar disorder	0.294	0.865	0.541
Post-traumatic stress disorder	0.309	0.117	0.541
Panic disorder	0.688	0.833	0.688
Obsessive–compulsive disorder	0.397	0.456	0.556
Anorexia nervosa	0.023	0.304	0.081

Adjusted *p*-values were obtained using the FDR multiple correction based on the *p*-values from IVW method.

### 3.2. The effects of mental disorders on ER+ and ER− breast cancer

On the basis of GWAS dataset from ER+ and ER- breast cancer, we conducted two-sample MR studies to determine whether psychiatric disorders were associated with a higher risk of different pathological subtypes of breast cancer. For GWAS data of exposures from the PGC, schizophrenia significantly increased the risks of both ER+ (OR 1.05, 95%CI [1.02–1.07], *p* = 5.94 × 10^−5^) and ER− (OR 1.04, 95%CI [1.01–1.07], *p* = 0.006) breast cancer based on the IVW analyses. The results of Bonferroni and FDR multiple correction methods were consistent (Figure 1 and Table 1). Intriguingly, according to the results of Bonferroni multiple corrections, a suggestive statistical significance was revealed between major depressive disorder and the risk of ER- breast cancer (OR 1.15, 95%CI [1.01–1.30], *p* = 0.030), but there was no statistical significance for ER+ breast cancer (Figures 1 2). However, the results of the FDR method showed no significant causal relationships between major depressive disorder and both subtypes of breast cancer (Table 1). The results based on MR-Egger and Weighted median methods were essentially identical to those from IVW. Genetic instrumental variables did not exhibit any significant pleiotropy effects, as determined by the MR-Egger test (Supplementary Table S5). As a consequence of the leave-one-out analyses, it was determined that no instrumental variables significantly influenced the causal relationships between mental diseases and the two subtypes of breast cancer (Supplementary Figures S2, S3, S5, S6). According to multivariable MR analyses, only schizophrenia significantly contributed to the increase of ER- breast cancer (IVW: OR 1.06, 95%CI [1.02–1.10], *p* = 0.001; MR-Egger: OR 1.06, 95%CI [1.03–1.10], *p* = 0.001), whereas major depressive disorder did not play a significant role (Figure 3). The MR analyses further revealed that bipolar disorder, post-traumatic stress disorder, panic disorder, obsessive–compulsive disorder, and anorexia nervosa did not significantly alter the risks of ER+ and ER- breast cancer (Figure 1 and Supplementary Table S5). In addition, Supplementary Table S4 showed the results of LDSC regression analyses between mental disorders and the two subtypes of breast cancer. Nevertheless, when the GWAS data of mental disorders were derived from the UK Biobank, schizophrenia could not significantly increased the risks of both subtypes (Supplementary Tables S6, S7). The causal relationships between seven psychiatric disorders and prostate cancer risk were demonstrated in Supplementary Table S8.

### 3.3. The effects of mental disorders on breast benign tumors and breast inflammatory diseases

As illustrated in Figure 1, none of the seven psychiatric disorders had significant impacts on risks of breast benign tumors and breast inflammatory diseases when using the IVW approach (Figure 1). Furthermore, both MR-Egger and Weighted median methods reached similar conclusions. Neither heterogeneity nor pleiotropy was observed according to the results (Supplementary Tables S9, S10).

## 4. Discussion

Our study investigate the causal links between seven psychiatric disorders and the risks of breast cancer, breast benign tumors, and breast inflammatory diseases using MR analyses for the first time. When the GWAS data for psychiatric disorders were derived from the PGC, schizophrenia and major depressive disorder were demonstrated to significantly increase the risk of overall breast cancer based on the two-sample MR analyses, in line with previous researches (16, 37). We stratified the analyses based on the ER status of breast cancer, since the pathogenesis and responses to treatments differ for each pathological state. It was demonstrated that schizophrenia was positively associated with both ER+ and ER- breast cancer risks, whereas major depressive disorder was not. Nevertheless, only schizophrenia, and not major depressive disorder, significantly elevated the risks of both overall and ER- breast cancer in multivariable MR analyses. Additionally, the seven psychiatric disorders were not significantly related to breast benign tumors or breast inflammatory diseases. Surprisingly, schizophrenia could not significantly increased the risk of breast cancer when we used the GWAS data of mental disorders from the the UK Biobank.

This study provided some insights for the correlation between schizophrenia and breast cancer. The prevalence of breast cancer has been demonstrated to be higher among people suffering from severe mental disorders than in the general population, according to previous epidemiological studies (38, 39). One meta-analysis, which included seven studies on breast cancer incidence in patients with schizophrenia, concluded that there was a 12% increased risk (40). In 1996, a small cross-sectional study of patients with chronic psychoses demonstrated that the prevalence of breast cancer was nearly 9.5 times higher than in the general population (41). Several other studies have also discovered higher rates of breast cancer in people with schizophrenia (10–12, 42).

Several possible factors may contribute to the connection between schizophrenia and breast cancer. Data from a Swedish research suggested an increased risk of breast cancer after the diagnosis of schizophrenia attributed to the use of antipsychotic drugs (43). Antipsychotics, particularly risperidone, have been known to increase prolactin, a neuroendocrine hormone that can be elevated during pregnancy and lactation (44). A recent cross-sectional epidemiological research documented hyperprolactinemia in over half of the subjects taking atypical antipsychotics (45). There were evidences that prolactin levels were associated with breast cancer risk, regardless of whether the patient was pre-or post-menopausal (46–49). There has been extensive reporting regarding the genetic traits shared by schizophrenia and breast cancer during recent years (50). Genome-wide association studies of schizophrenia revealed the genetic background similar to that of breast cancer, including some immune traits and genetic overlaps (51, 52). Researchers reported a positive link in the phenotypic and genetic between schizophrenia and breast cancer, estimating the percentage of gene overlap at 0.14, and identified a shared locus (GATA-D2A) at 19p13 as a significant risk factor for the development of these two diseases (52). Additionally, schizophrenia also leads to the degenerative change at the level of brain networks (53). In recent years, the epidemiological evidences of the association between neurodegenerative diseases and cancers has been emerging. Whereas the characteristic brain pathological changes of neurodegenerative diseases could lead to neuronal cell death and neurodegeneration, cancers were primarily determined by the process of infinite cell proliferation (54, 55). There were evidences that the common biological mechanisms to both diseases, such as oxidative stress, metabolic disorders, and inflammation, promoted not only cell apoptosis but also proliferation (56–58). Some of the immunometabolic markers observed in patients with schizophrenia might also contribute to the development of breast cancer (59–61). Inflammatory changes occured in schizophrenia patients could also be flagged as cancer risk factors (62). It was demonstrated that elevated levels of IL-33 in the serum not only appeared in schizophrenia patients (63), but also indicated breast cancer recurrence (64–66). In this study, MR analyses using data from the PGC and the UK Biobank produced inconsistent results, suggesting that the relationship between schizophrenia and breast cancer may be due to the underlying horizontal pleiotropy leading to false positive results, rather than a true causal relationship.

This study revealed significant positive association between major depressive disorder and overall breast cancer risk in the two-sample MR analysis, but not with the multivariable MR analysis. Several previous two-sample MR studies have reported an increased risk for breast cancer associated with major depressive disorder (19, 37), However, previous studies did not perform multivariable MR analyses and account for genetic links among multiple psychiatric disorders. Several observational studies have suggested that major depressive disorder might be associated with an increased risk of breast cancer (6–8), other studies, however, have found no such conclusion (67–69). Possibly, it might stem from the wide variations in study settings, including confounding factors, that these controversies occurred. Prior studies have also linked post-traumatic stress disorder and bipolar disorder to breast cancer. It was noted in 2022 that a number of breast cancer patients exhibited symptoms similar to those of post-traumatic stress disorder, which were thought to be mediated by chronic inflammation, such as NF-κB, AKt, p53, and other inflammatory pathways (70). Another MR analysis indicated that bipolar disorder was causally linked to a higher breast cancer rate (71). On the one hand, the reason why our conclusion differed from previous ones might be that the sample size of GWAS data was not large enough to produce statistical differences, on the other hand, previous results might be affected by a variety of biases or confounding factors. However, randomized controlled trials are still the best way to determine exactly how these mental illnesses link to breast cancer.

Mental illnesses have been studied less extensively in relation to breast benign tumors and breast inflammatory diseases. Researchers described a benign intraductal papilloma in a male patient with a history of chronic mental illness who had been treated with phenothiazines for a long time, considering that phenothiazines induced elevated serum prolactin levels so as to promote the development of the breast benign tumor (72). Similarly, another male with chronic schizophrenia who had been treated with phenothiazines for a long period was diagnosed as the breast cystic tumor (73). These cases are rare and insufficient to provide sufficient evidences. Our study was the first to examine the causal correlations between psychiatric disorders and the risks of breast benign tumors and breast inflammatory diseases, with the negative results. Nevertheless, the sample sizes of these two diseases were small, more studies with larger sample sizes are still required for strong evidences.

In this study, several advantages are apparent. First of all, since the alleles formed gametes on a random basis during meiosis, confounding factors could not skew the causal associations between genotype and diseases during MR analyses, which had been a major limitation of traditional observational researches. Furthermore, public data were easier to be obtained in MR analyses compared with prospective cohort studies or randomized controlled trials, reducing the time and expenses required for researches. Third, unlike previous MR analyses related to mental diseases, this study took into account the multivariable MR analyses due to the genetic correlation between schizophrenia and major depressive disorder. In spite of this, we must acknowledge a few limitations of our analyses. At first, the different results from the two databases in this study might be due to the false positive results generated by the horizontal pleiotropy. In addition, GWAS data were derived from European population, so that generalizations of the results should be validated in other ethnicities. Finally, The GWAS data of these seven mental disorders in this study had different publication time and sample sizes, we could not unify the criteria screening SNPs strongly associated with exposures, therefore, there might be some heterogeneity in the results. More data with the same sample size and publication time should be included in the future to explore the relationships between mental disorders and breast diseases.

Overall, the association between schizophrenia and breast cancer in this study may be false positive result due to the underlying horizontal pleiotropy, rather than a causal relationship. There were also insufficient evidences that mental disorders and the other breast diseases were causally related. More prospective experimental designs, such as randomized controlled trials or cohort studies, will be necessary in the future to demonstrate more accurate results.

### Novelty and impact

Previous observational studies have demonstrated the associations between some psychiatric disorders and breast cancer, however, it is unclear whether mental illnesses affect breast benign tumors and breast inflammatory diseases. This study was the first to explore relationships between seven psychiatric disorders and the risks of overall breast cancer, two subtypes (ER+ and ER-), breast benign tumors and breast inflammatory diseases using Mendelian randomization analyses.

Our study found some associations between schizophrenia and breast cancer, but it might be false positive results due to underlying horizontal pleiotropy, and there were no evidences of causal relationships between psychiatric disorders and these breast diseases.

## Data availability statement

The datasets presented in this study can be found in online repositories. The names of the repository/repositories and accession number(s) can be found in the article/Supplementary material.

## Ethics statement

Only publicly available GWAS data were used in this study, and the ethics approval could be available in the original GWAS studies.

## Author contributions

XinW and XiaW designed the study. SZ, CY, KF, JL, XK, and RZ were responsible for data extraction and collation. QS was responsible for statistical analyses. FR wrote the manuscript. All authors contributed to the article and approved the submitted version.

## Funding

This research was supported by the National Key Research and Development Program of China (Grant No. 2019YFE0110000), National Natural Science Foundation of China (Grant No. 82072097), CAMS Innovation Fund for Medical Science (CIFMS) (Grant No. 2020-I2M-C&T-B-069, 2021-I2M-1-014), Beijing Hope Run Special Fund of Cancer Foundation of China (Grant No. LC2020A18).

## Conflict of interest

The authors declare that the research was conducted in the absence of any commercial or financial relationships that could be construed as a potential conflict of interest.

## Publisher’s note

All claims expressed in this article are solely those of the authors and do not necessarily represent those of their affiliated organizations, or those of the publisher, the editors and the reviewers. Any product that may be evaluated in this article, or claim that may be made by its manufacturer, is not guaranteed or endorsed by the publisher.

## Footnotes

^1^ https://gwas.mrcieu.ac.uk/

^2^ http://www.nealelab.is/uk-biobank

## Abbreviations

ER: estrogen receptor
GWAS: genome-wide association study
PGC: Psychiatric Genomics Consortium
SNPs: single-nucleotide polymorphisms
BCAC: Breast Cancer Association Consortium
MR: Mendelian randomization
SIR: standardized incidence rates
EAF: effector allele frequency
IVW: inverse variance weighted
FDR: false discovery rate
LOO: leave-one-out
LDSC: linkage disequilibrium score regression
