# Supplementary material for: An exploration of the correlations between seven psychiatric disorders and the risks of breast cancer, breast benign tumors and breast inflammatory diseases: Mendelian randomization analyses
Source: Front Psychiatry. 2023 Jun 28;14:1179562. doi: 10.3389/fpsyt.2023.1179562 (PMC10338175; doi:10.3389/fpsyt.2023.1179562)
Supplement: Supplementary file 1 [file Data_Sheet_1.docx]

**Supplementary Table 1** Detailed information on GWAS data for seven psychiatric disorders from the PGC

| **Psychiatric disorders** | **Year** | **PMID** | **Source** |
| --- | --- | --- | --- |
| Schizophrenia | 2022 | 35396580 | Trubetskoy V, et al. 2022, Nature |
| Major depressive disorder | 2019 | 30718901 | Howard DM, et al. 2019, Nature neuroscience |
| Bipolar disorder | 2021 | 34002096 | Mullins N, et al. 2021, Nature genetics |
| Post-traumatic stress disorder | 2019 | 31594949 | Nievergelt CM, et al. 2019, Nature communications |
| Panic disorder | 2019 | 31712720 | Forstner AJ, et al. 2021, Molecular psychiatry |
| Obsessive-compulsive disorder | 2018 | 28761083 | IOCDF-GC and OCGAS. 2018, Molecular psychiatry |
| Anorexia nervosa | 2017 | 28494655 | Duncan L, et al. 2017, The American journal of psychiatry |

**Supplementary** **Table 2** Detailed information on GWAS data for breast diseases

| **Outcomes** | **Year** | **Sample Size** | **Link** |
| --- | --- | --- | --- |
| Breast cancer | 2017 | 228951 | https://gwas.mrcieu.ac.uk/datasets/ieu-a-1126/ |
| ER+ Breast cancer | 2017 | 175475 | https://gwas.mrcieu.ac.uk/datasets/ieu-a-1127/ |
| ER- Breast cancer | 2017 | 127442 | https://gwas.mrcieu.ac.uk/datasets/ieu-a-1128/ |
| Breast benign tumors | 2021 | 103153 | https://gwas.mrcieu.ac.uk/datasets/finn-b-CD2_BENIGN_BREAST_EXALLC/ |
| Breast inflammatory diseases | 2021 | 115787 | https://gwas.mrcieu.ac.uk/datasets/finn-b-N14_INFLAMMBREAST/ |

**Supplementary Table 3** Links of GWAS data for seven psychiatric disorders from the UK biobank

| **Psychiatric disorders** | **Link** |
| --- | --- |
| Schizophrenia | https://broad-ukb-sumstats-us-east-1.s3.amazonaws.com/round2/additive-tsvs/20002_1289.gwas.imputed_v3.both_sexes.tsv.bgz |
| Major depressive disorder | https://broad-ukb-sumstats-us-east-1.s3.amazonaws.com/round2/additive-tsvs/20126_3.gwas.imputed_v3.both_sexes.tsv.bgz |
| Bipolar disorder | https://broad-ukb-sumstats-us-east-1.s3.amazonaws.com/round2/additive-tsvs/20122.gwas.imputed_v3.both_sexes.tsv.bgz |
| Post-traumatic stress disorder | https://broad-ukb-sumstats-us-east-1.s3.amazonaws.com/round2/additive-tsvs/20002_1469.gwas.imputed_v3.both_sexes.tsv.bgz |
| Panic disorder | https://broad-ukb-sumstats-us-east-1.s3.amazonaws.com/round2/additive-tsvs/F5_PANIC.gwas.imputed_v3.both_sexes.tsv.bgz |
| Obsessive-compulsive disorder | https://broad-ukb-sumstats-us-east-1.s3.amazonaws.com/round2/additive-tsvs/20544_7.gwas.imputed_v3.both_sexes.tsv.bgz |
| Anorexia nervosa | https://broad-ukb-sumstats-us-east-1.s3.amazonaws.com/round2/additive-tsvs/20544_16.gwas.imputed_v3.both_sexes.tsv.bgz |

**Supplementary Table 4** The results of LDSC regression analyses

|  | **Overall breast cancer** | |  | **ER+ breast cancer** | |  | **ER- breast cancer** | |
| --- | --- | --- | --- | --- | --- | --- | --- | --- |
| **Psychiatric disorders** | **Genetic correlation** | ***P*-value** |  | **Genetic correlation** | ***P*-value** |  | **Genetic correlation** | ***P*-value** |
| Schizophrenia | 0.132 | 3.16×10^-10^ |  | 0.110 | 2.57×10^-6^ |  | 0.087 | 0.003 |
| Major depressive disorder | 0.089 | 4.98×10^-5^ |  | 0.072 | 0.003 |  | 0.089 | 0.009 |
| Bipolar disorder | 0.080 | 3.22×10^-4^ |  | 0.063 | 0.008 |  | 0.044 | 0.180 |
| Post-traumatic stress disorder | 0.238 | 0.437 |  | 0.319 | 0.319 |  | -0.185 | 0.729 |
| Panic disorder | 0.087 | 0.099 |  | 0.093 | 0.111 |  | 0.021 | 0.786 |
| Obsessive-compulsive disorder | -0.114 | 0.185 |  | -0.062 | 0.522 |  | -0.207 | 0.118 |
| Anorexia nervosa | 0.055 | 0.233 |  | 0.053 | 0.284 |  | 0.036 | 0.596 |

**Supplementary Table 5** The effects of psychiatric disorders on ER+ and ER- breast cancer risks from MR Egger and Weighted median methods

| **Psychiatric disorders** | **ER-positive** | | | | | | |  | **ER-negative** | | | | | | |
| --- | --- | --- | --- | --- | --- | --- | --- | --- | --- | --- | --- | --- | --- | --- | --- |
|  | **MR Egger** | |  | **Weighted median** | | ***P*****_heterogeneity_** | ***P*_pleiotropy_** |  | **MR Egger** | |  | **Weighted median** | | ***P*_heterogeneity_** | ***P*_pleiotropy_** |
|  | **OR(95% CI)** | ***P*-value** |  | **OR(95% CI)** | ***P*-value** |  |  |  | **OR(95% CI)** | ***P*-value** |  | **OR(95% CI)** | ***P*-value** |  |  |
| Schizophrenia | 1.11(1.03-1.20) | 8.31×10^-3^ |  | 1.05(1.02-1.08) | 2.46×10^-3^ | 2.01×10^-5^ | 0.114 |  | 0.98(0.89-1.08) | 0.708 |  | 1.03(0.99-1.08) | 0.101 | 0.008 | 0.208 |
| Major depressive disorder | 0.86(0.51-1.44) | 0.567 |  | 1.01(0.90-1.14) | 0.827 | 0.005 | 0.441 |  | 1.18(0.63-2.22) | 0.609 |  | 1.13(0.95-1.34) | 0.175 | 0.136 | 0.927 |
| Bipolar disorder | 0.85(0.68-1.05) | 0.132 |  | 0.96(0.92-1.01) | 0.152 | 0.067 | 0.215 |  | 1.07(0.81-1.40) | 0.650 |  | 0.97(0.90-1.04) | 0.414 | 0.251 | 0.570 |
| Post-traumatic stress disorder | 1.02(0.91-1.14) | 0.702 |  | 1.03(0.97-1.10) | 0.281 | 0.487 | 0.868 |  | 1.10(0.93-1.31) | 0.289 |  | 1.05(0.94-1.16) | 0.376 | 0.654 | 0.524 |
| Panic disorder | 1.01(0.94-1.07) | 0.835 |  | 1.03(0.99-1.07) | 0.167 | 0.287 | 0.671 |  | 1.01(0.92-1.10) | 0.893 |  | 1.02(0.97-1.08) | 0.457 | 0.907 | 0.655 |
| Obsessive-compulsive disorder | 0.97(0.91-1.04) | 0.447 |  | 1.01(0.98-1.04) | 0.576 | 0.638 | 0.179 |  | 0.97(0.86-1.09) | 0.625 |  | 0.96(0.91-1.02) | 0.162 | 0.186 | 0.944 |
| Anorexia nervosa | 1.00(0.91-1.10) | 0.959 |  | 0.98(0.94-1.03) | 0.526 | 0.659 | 0.541 |  | 0.94(0.82-1.07) | 0.364 |  | 1.01(0.94-1.09) | 0.725 | 0.553 | 0.355 |

**Supplementary Table 6** The *P* values for MR analyses of psychiatric disorders and ER+ breast cancer risk (GWAS

data for exposures were derived from the UK Biobank)

| **Psychiatric disorders** | ***P*-value by IVW** | ***P*-value by MR Egger** | **Adjusted *P-*value** |
| --- | --- | --- | --- |
| Schizophrenia | 0.599 | 0.497 | 0.997 |
| Major depressive disorder | 0.049 | 0.625 | 0.172 |
| Bipolar disorder | 0.997 | 0.858 | 0.997 |
| Post-traumatic stress disorder | 0.012 | 0.012 | 0.084 |
| Panic disorder | 0.881 | 0.995 | 0.997 |
| Obsessive-compulsive disorder | 0.882 | 0.909 | 0.997 |
| Anorexia nervosa | 0.207 | 0.624 | 0.483 |

*Adjusted* *P-values were* [*obtain*](javascript:;)*ed using the FDR multiple correction based on the P-values from IVW method.*

**Supplementary Table 7** The *P* values for MR analyses of psychiatric disorders and ER- breast cancer risk (GWAS

data for exposures were derived from the UK Biobank)

| **Psychiatric disorders** | ***P*-value by IVW** | ***P*-value by MR Egger** | **Adjusted *P-*value** |
| --- | --- | --- | --- |
| Schizophrenia | 0.395 | 0.476 | 0.861 |
| Major depressive disorder | 0.713 | 0.652 | 0.861 |
| Bipolar disorder | 0.861 | 0.786 | 0.861 |
| Post-traumatic stress disorder | 0.655 | 0.851 | 0.861 |
| Panic disorder | 0.306 | 0.344 | 0.861 |
| Obsessive-compulsive disorder | 0.750 | 0.712 | 0.861 |
| Anorexia nervosa | 0.702 | 0.228 | 0.861 |

*Adjusted P-values were* [*obtain*](javascript:;)*ed using the FDR multiple correction based on the P-values from IVW method.*

**Supplementary Table 8** The effects of psychiatric disorders on prostate cancer risk from IVW method

| **Psychiatric disorders** | **OR(95% CI)** | ***P*-value** |
| --- | --- | --- |
| Schizophrenia | 1.005(1.003-1.007) | 9.03×10^-6^ |
| Major depressive disorder | 0.999(0.991-1.006) | 0.715 |
| Bipolar disorder | 1.000(0.996-1.004) | 0.907 |
| Post-traumatic stress disorder | 0.997(0.992-1.002) | 0.228 |
| Panic disorder | 0.998(0.995-1.001) | 0.226 |
| Obsessive-compulsive disorder | 0.999(0.996-1.002) | 0.350 |
| Anorexia nervosa | 1.001(0.998-1.004) | 0.426 |

*The prostate cancer GWAS data were from the UK Biobank, and were obtained from the MRC*

*Integrative Epidemiology Unit (https://gwas.mrcieu.ac.uk/), the GWAS ID of which was ieu-b-4809.*

**Supplementary Table** **9** The effects of psychiatric disorders on the risks of breast benign tumors from MR Egger and

Weighted median methods

| **Psychiatric disorders** | **MR Egger** | |  | **Weighted median** | | ***P*_heterogeneity_** | ***P*_pleiotropy_** |
| --- | --- | --- | --- | --- | --- | --- | --- |
|  | **OR(95% CI)** | ***P*-value** |  | **OR(95% CI)** | ***P*-value** |  |  |
| Schizophrenia | 1.23(0.96-1.56) | 0.099 |  | 1.09(0.97-1.22) | 0.150 | 0.183 | 0.158 |
| Major depressive disorder | 1.62(0.28-9.40) | 0.594 |  | 1.21(0.79-1.86) | 0.371 | 0.571 | 0.781 |
| Bipolar disorder | 0.52(0.25-1.06) | 0.077 |  | 0.98(0.81-1.20) | 0.878 | 0.377 | 0.077 |
| Post-traumatic stress disorder | 0.86(0.65-1.15) | 0.326 |  | 0.98(0.80-1.21) | 0.873 | 0.781 | 0.214 |
| Panic disorder | 1.00(0.76-1.31) | 0.987 |  | 1.01(0.89-1.14) | 0.891 | 0.334 | 0.735 |
| Obsessive-compulsive disorder | 1.03(0.79-1.34) | 0.837 |  | 0.97(0.85-1.10) | 0.620 | 0.557 | 0.965 |
| Anorexia nervosa | 1.15(0.83-1.59) | 0.424 |  | 1.07(0.89-1.28) | 0.469 | 0.208 | 0.500 |

**Supplementary Table 10** The effects of psychiatric disorders on the risks of breast inflammatory diseases from MR Egger and Weighted median methods

| **Psychiatric disorders** | **MR Egger** | |  | **Weighted median** | | ***P*_heterogeneity_** | ***P*_pleiotropy_** |
| --- | --- | --- | --- | --- | --- | --- | --- |
|  | **OR(95% CI)** | ***P*-value** |  | **OR(95% CI)** | ***P*-value** |  |  |
| Schizophrenia | 1.52(1.04-2.21) | 0.031 |  | 1.20(1.01-1.43) | 0.035 | 0.462 | 0.096 |
| Major depressive disorder | 0.53(0.03-10.50) | 0.681 |  | 0.82(0.40-1.68) | 0.595 | 0.321 | 0.681 |
| Bipolar disorder | 1.32(0.42-4.21) | 0.635 |  | 0.88(0.64-1.21) | 0.426 | 0.912 | 0.575 |
| Post-traumatic stress disorder | 1.33(0.83-2.11) | 0.248 |  | 0.93(0.66-1.31) | 0.671 | 0.819 | 0.117 |
| Panic disorder | 1.00(0.69-1.45) | 0.985 |  | 1.05(0.87-1.28) | 0.589 | 0.952 | 0.659 |
| Obsessive-compulsive disorder | 0.79(0.49-1.27) | 0.359 |  | 1.00(0.81-1.24) | 0.990 | 0.274 | 0.510 |
| Anorexia nervosa | 0.69(0.44-1.09) | 0.137 |  | 0.89(0.69-1.14) | 0.354 | 0.813 | 0.165 |


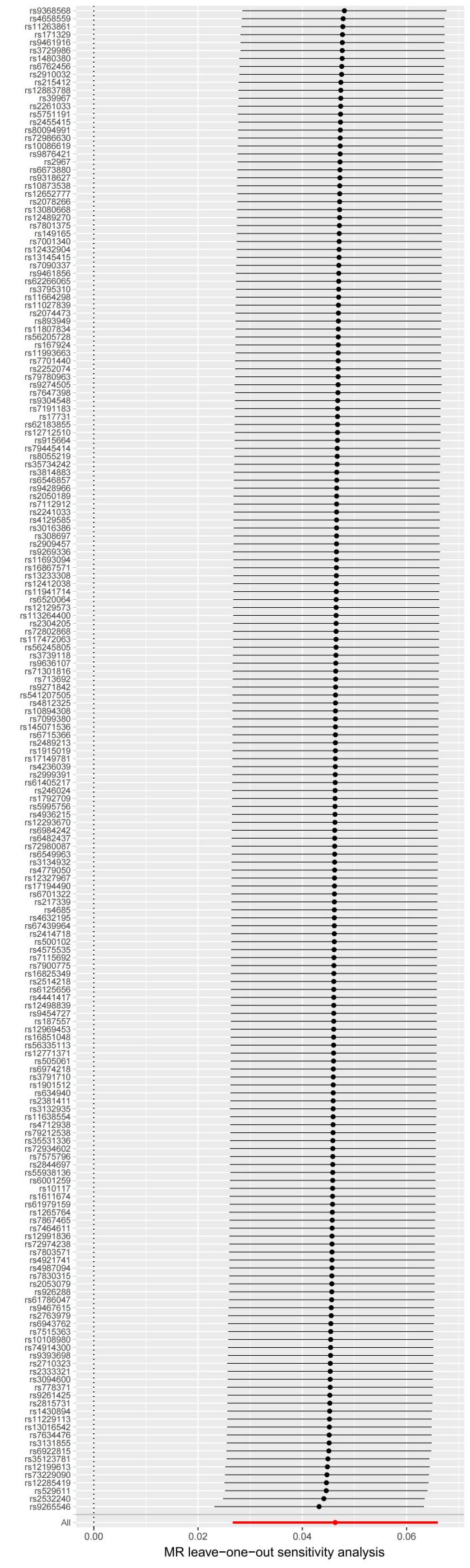


**Supplementary Figure 1**

Leave-one-out analysis of schizophrenia and overall breast cancer risk.


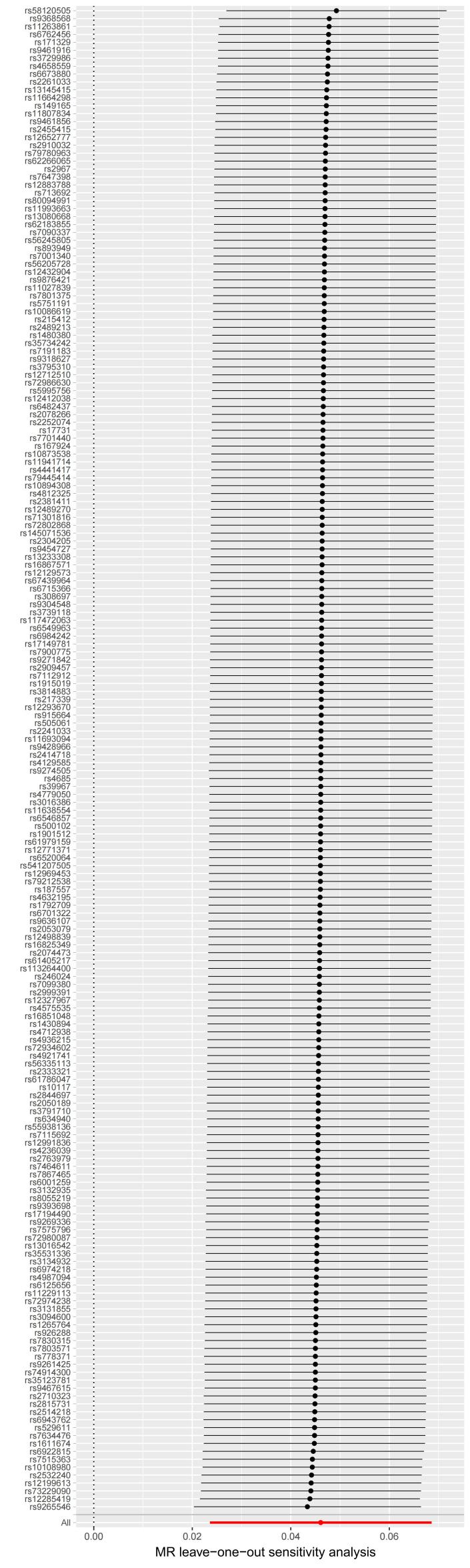


**Supplementary Figure 2**

Leave-one-out analysis of schizophrenia and ER+ breast cancer risk.

**
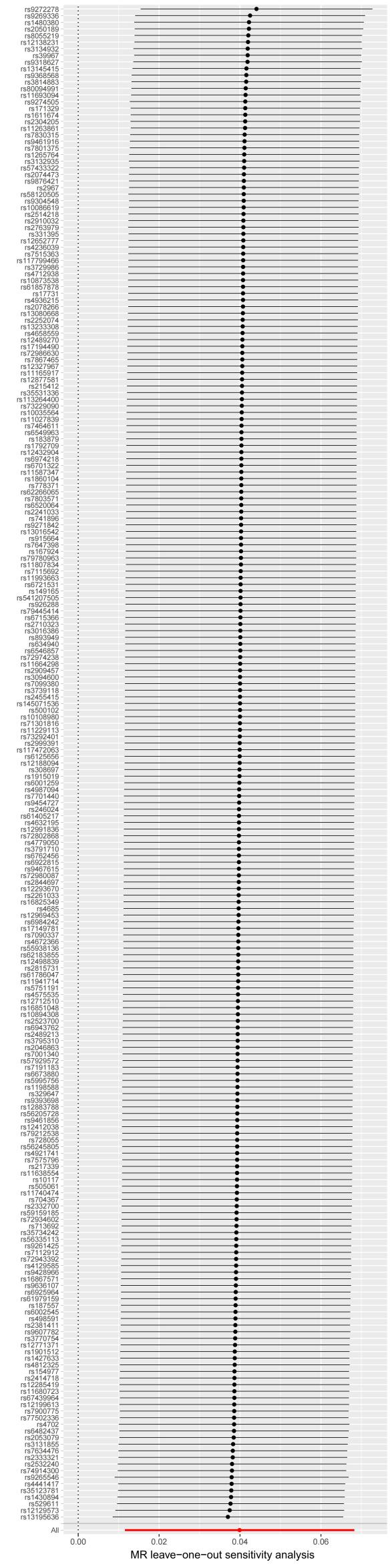
**

**Supplementary Figure 3**

Leave-one-out analysis of schizophrenia and ER- breast cancer risk.


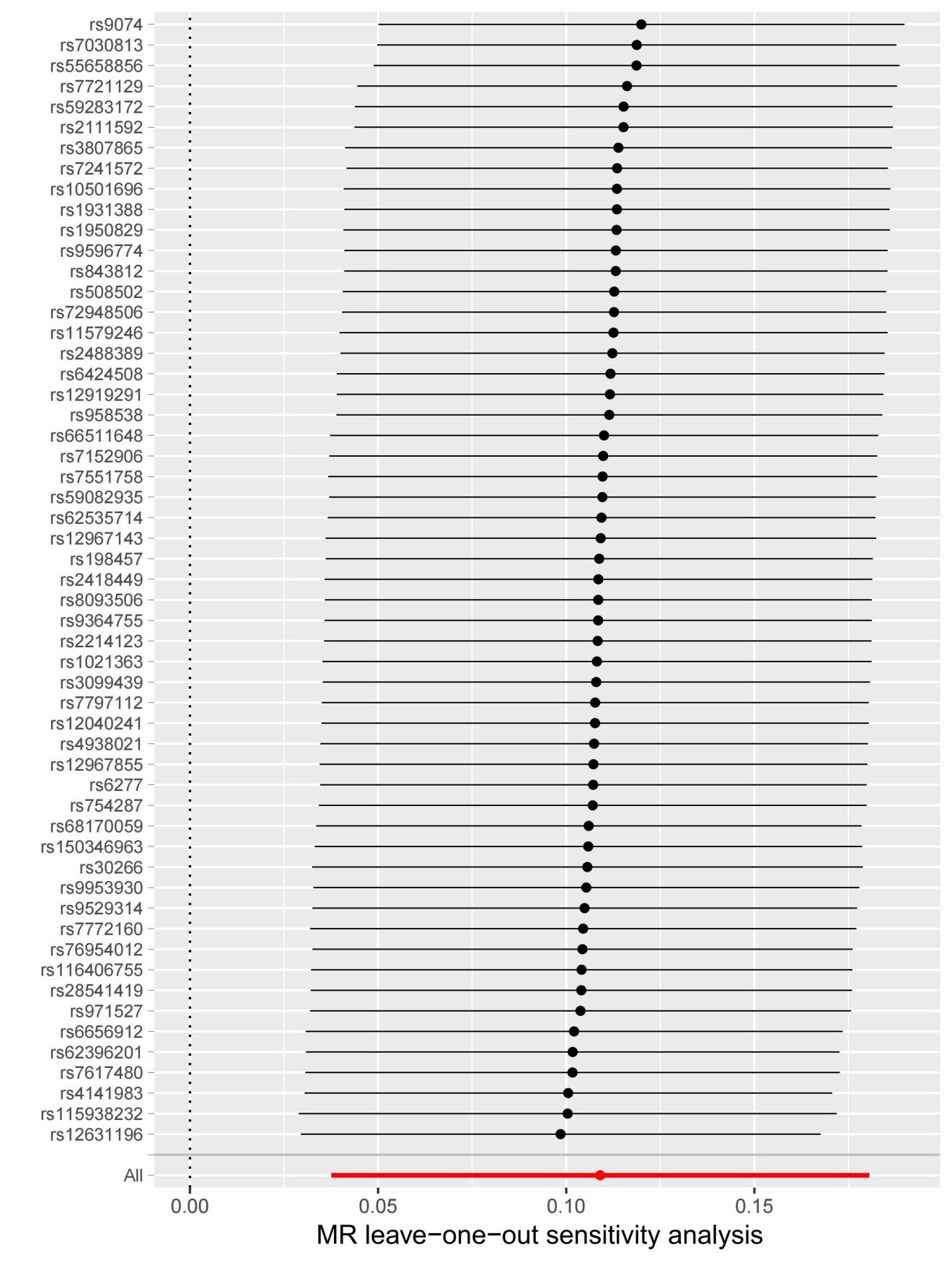


**Supplementary Figure 4** Leave-one-out analysis of major depressive disorder and overall breast cancer risk.


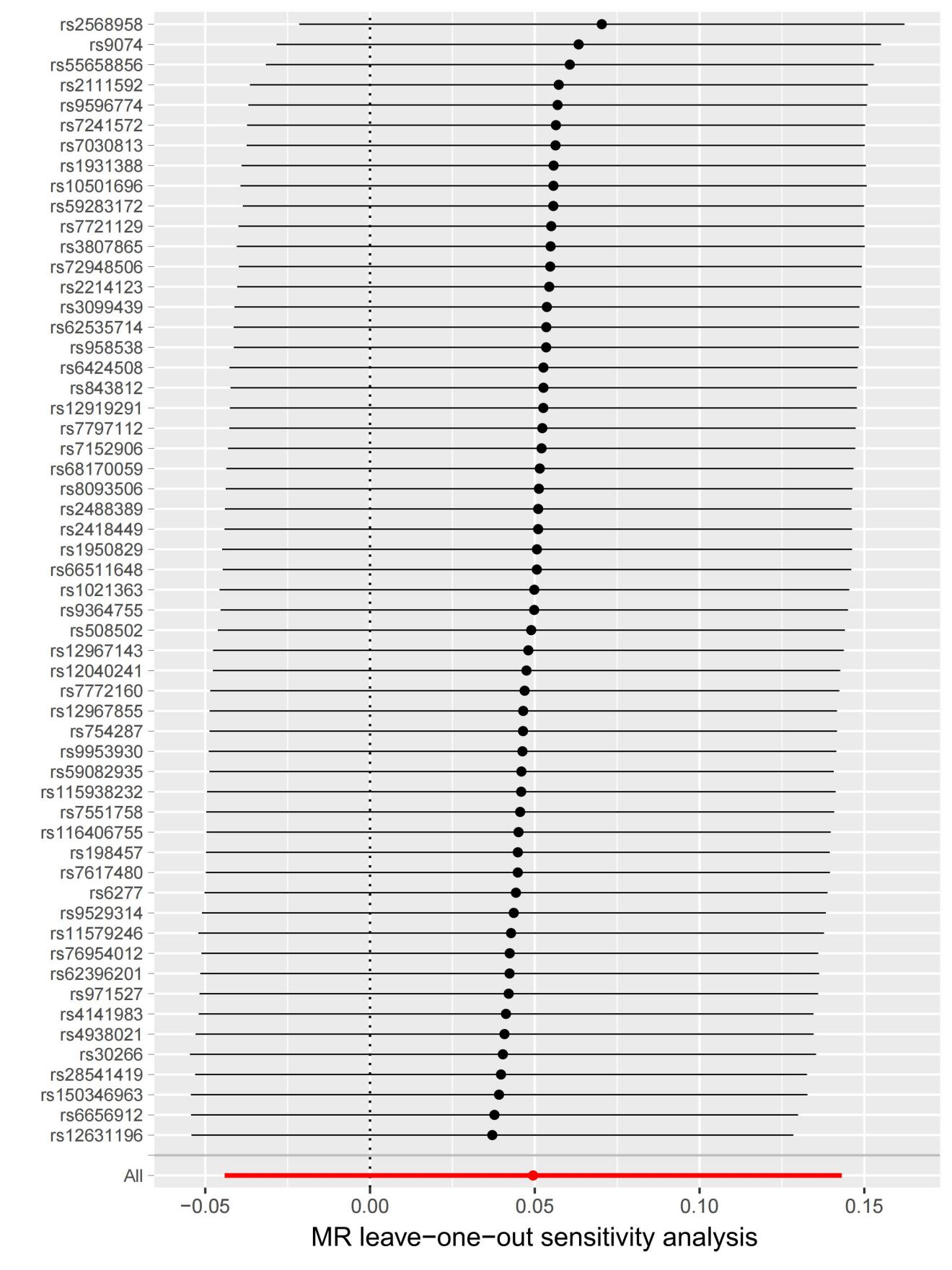


**Supplementary Figure 5** Leave-one-out analysis of major depressive disorder and ER+ breast cancer risk.


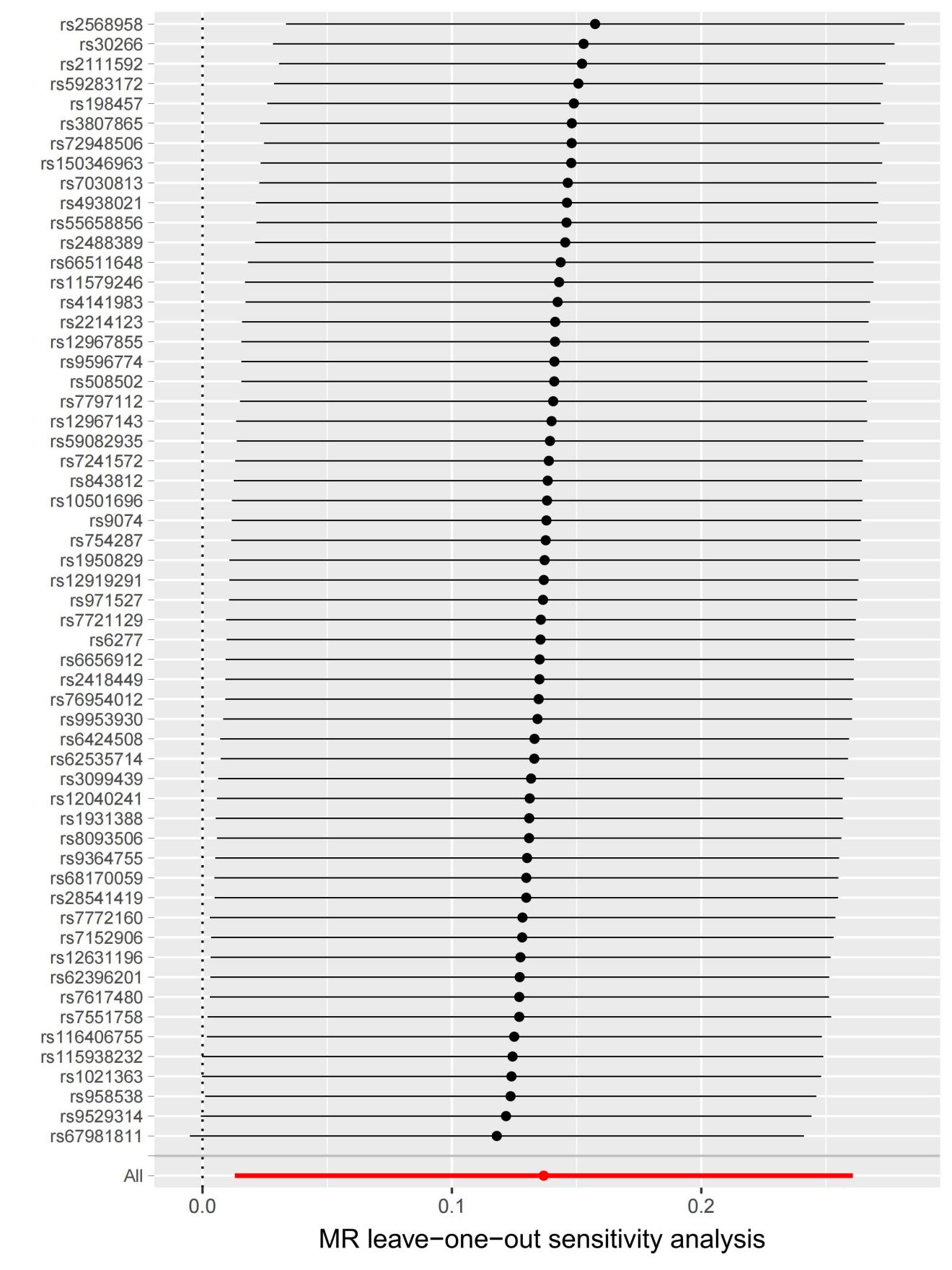


**Supplementary Figure 6** Leave-one-out analysis of major depressive disorder and ER- breast cancer risk.
